# Congenital Long QT Syndrome Masquerading as Epilepsy: A Case Report

**DOI:** 10.7759/cureus.73590

**Published:** 2024-11-13

**Authors:** Abdulrahman Irhouma, Ahmed Ziada, Yusif Shakanti, Nicholas Jenkins

**Affiliations:** 1 General Medicine, Manchester University NHS Foundation Trust, Manchester, GBR; 2 Cardiology, Manchester University NHS Foundation Trust, Manchester, GBR; 3 Internal Medicine, East Cheshire NHS Foundation Trust, Macclesfield, GBR

**Keywords:** ecg, icd, long qt, loss of consciousness, seizures, tdp, torsades de pointes

## Abstract

In this case report, we present a 24-year-old woman with a previous diagnosis of epilepsy who was admitted to the hospital following loss of consciousness (LOC). It was initially assumed that this was an epileptic seizure based on her previous diagnosis of epilepsy; however, a review of her electrocardiograms (ECGs) revealed a prolonged QT interval. She was admitted to the cardiology ward for continuous ECG monitoring and subsequently developed self-limiting torsades de pointes (TDP). A diagnosis of congenital long QT syndrome (LQTS) was established, her anticonvulsant treatment was withdrawn, and she was managed with nadolol, mexiletine, and an implantable cardioverter defibrillator (ICD). This case underscores the importance of excluding cardiac disease with secondary anoxic seizures in patients with apparent epilepsy and in particular the need for all patients to have a baseline 12-lead ECG as part of their initial assessment.

## Introduction

Congenital long QT syndrome (LQTS) is a dominantly inherited genetic disorder, arising from a mutation in a number of potential cardiac ion channel genes, which can cause syncope and cardiac arrest due to torsades de pointes (TDP), a life-threatening ventricular arrhythmia [1,2].

Clinically, LQTS can present with a wide range of symptoms, including syncope, palpitations, and, in some cases, anoxic seizure-like episodes [3]. This can lead to diagnostic challenges; patients may be erroneously diagnosed with epilepsy due to the seizure-like nature of their symptoms, as is described in this case. This can result in inappropriate treatments, including the use of anti-epileptic medications that do not address and sometimes exacerbate the underlying cardiac condition through means of further QT prolongation, thus increasing the risk of life-threatening arrhythmias [4].

The case we present underscores the necessity for thorough clinical evaluation, including taking a detailed family history and 12-lead electrocardiogram (ECG), when presented with patients with apparent epilepsy or exhibiting unexplained loss of consciousness (LOC) or convulsive movements. The early recognition of LQTS is crucial because of the life-threatening nature of the condition, the effective treatment strategies available, and the urgent need to screen other family members [5].

## Case presentation

We report a case of congenital LQTS masquerading as epilepsy in a 24-year-old woman with no other comorbidities. She attended the emergency department (ED) following an increased frequency of LOC over the previous four days. She had had a normal vaginal delivery five weeks previously with no complications, and she took levetiracetam 1 g twice daily for her epilepsy, which was diagnosed seven years ago. The episodes of LOC were described as short periods of "blackouts," usually less than one minute, preceded by an aura of blurred vision and ringing in her ears. On admission, she had a plasma sodium of 141 mmol/L, a potassium of 3.6 mmol/L, and a magnesium of 0.75 mmol/L. There was no post-ictal period, no tongue biting, and no incontinence. This was initially treated as an increased frequency of her epileptic seizures.

**Figure 1:**
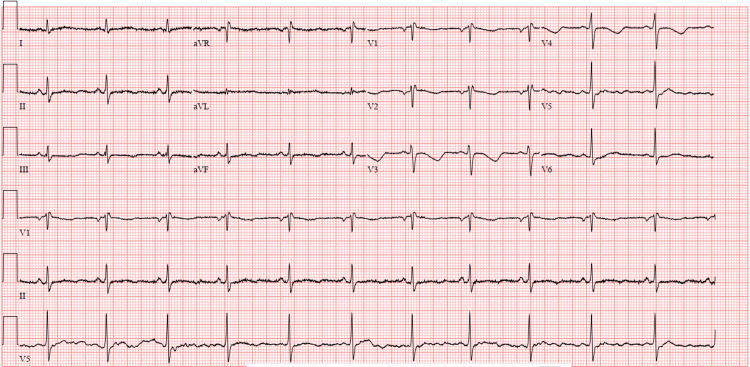
ECG showing QT prolongation and inverted T waves. Paper speed: 25 mm/second. ECG: electrocardiogram

Following this, she had a further episode of LOC on the ward, which self-terminated. Her ECG showed a QT/corrected QT (QTc) segment of 522/559 ms (despite electrolytes being within range) with T wave inversion across the precordial leads (Figure 1).

On further questioning, she complained that her episodes of LOC were almost always preceded by palpitations. She also reported that her sister had suddenly passed away at the age of 21 after waking up from sleep and collapsing with no warning signs. Her sister also had a similar diagnosis of epilepsy.

She was admitted to the cardiology ward for continuous ECG monitoring. She was also reviewed by the neurology service, who agreed that she did not have epilepsy and recommended tapering her levetiracetam down over three days to ascertain whether this was a case of congenital LQTS or whether her long QT was drug-induced.

Later that day, her ECG monitor showed polymorphic ventricular tachycardia (in the form of TDP) lasting 6.1 seconds. She was started on nadolol 80 mg once daily and was given intravenous magnesium and potassium, aiming for a magnesium level of 1.0 mmol/L and potassium of 4.5 mmol/L. Further ECGs showed QTc intervals of up to 678 ms despite electrolyte optimization and the withdrawal of levetiracetam. She was initially suspected to have type 2 congenital LQTS. This prolongation of her QT was likely due to the slowing of the heart rate after the initiation of nadolol, so she was started on mexiletine 100 mg three times a day. She also had an echocardiogram, which showed a normal ventricular cavity size and mild left ventricular (LV) dysfunction with a visual ejection fraction (EF) of around 50% with no valvular abnormalities.

She had no further episodes of TDP or LOC following the initiation of nadolol and mexiletine, and she was counselled on the need for an implantable cardioverter defibrillator (ICD) as per the inherited cardiac disease multidisciplinary team recommendation. They also advised urgent ECG screening for the patient's siblings and her baby. Her mexiletine dose was increased to 500 mg daily, which achieved a QT interval of 481 ms. She had a dual-chamber ICD inserted with no complications and was discharged the following day.

She also had genetic testing, including the *CACNA1C*, *CALM1*, *CALM2*, *CALM3*, *KCNE1*, *KCNE2*, *KCNH2*, *KCNJ2*, *KCNQ1*, and *SCN5A* genes, and was not found to have any pathogenic variants. Three months later, she had not had any shocks from her device and had not had any further LOC episodes. A further extended genetic panel has been sent, and a cardiac magnetic resonance (CMR) has been requested due to the T wave inversion and borderline EF.

## Discussion

The presentation of LQTS as LOC can lead to a misdiagnosis of epilepsy, as illustrated by this case. Features commonly associated with epileptic seizures, such as aura-like symptoms preceding LOC, are also seen in LQTS, making it difficult to distinguish between the two initially [5]. The absence of post-ictal symptoms, tongue biting, or incontinence, however, raises suspicion for a non-epileptic cause of these events, as seen in this patient [6]. Additionally, the family history of her sister's sudden unexplained death is an important red flag [7]. She had not had a 12-lead ECG as part of her assessment in the epilepsy clinic.

The delayed diagnosis in this case reflects the difficulty in recognizing LQTS based solely on clinical presentation. Her persistently prolonged QTc interval on ECG was suggestive of congenital LQTS. The patient's persistently long QTc, despite the withdrawal of levetiracetam and electrolyte optimization, was indicative of an intrinsic disorder rather than a drug-induced one [4]. Additionally, her further report of palpitations prior to LOC episodes was a significant clue pointing to an arrhythmic cause. Thus, ECG remains a critical diagnostic tool in differentiating LQTS from epilepsy when seizure-like symptoms and LOC occur. A high index of suspicion, detailed family history, and careful evaluation of ECG are vital steps in the assessment of LOC.

Once identified, the management of congenital LQTS involves a multifaceted approach to reduce the risk of ventricular arrhythmias. This patient's treatment included beta-blockade with nadolol. This initially prolonged her QT interval further by means of slowing down her heart rate, and she was subsequently started on the antiarrhythmic drug mexiletine, which effectively stabilized her QTc interval and prevented further arrhythmic episodes. Beta-blockers are considered the first-line therapy for LQTS, reducing the incidence of arrhythmic events by blunting adrenergic stimulation [5]. The addition of mexiletine, which shortens the QT interval by blocking late sodium currents [8], is recommended in cases with persistently prolonged QTc intervals [9].

Given the high risk of sudden cardiac death (SCD) in LQTS patients, especially those with a history of syncope or arrhythmia, the patient was recommended for an ICD [3]. ICD implantation can provide lifesaving therapy in the event of life-threatening arrhythmias [10].

The genetic evaluation of this patient included testing for known LQTS-related genes, which returned negative results [7]. While a specific genetic mutation was not identified, this does not exclude LQTS, as approximately 20% of patients with clinically diagnosed LQTS have no identifiable mutation in known genes [11]. Family screening remains critical, as first-degree relatives may also carry a risk of LQTS even in the absence of a positive genetic finding [2]. Her siblings and newborn child were advised to undergo ECG screening, which is essential to identify at-risk individuals in families affected by inherited arrhythmic syndromes.

It is also important to recognize other cardiac causes of syncope, which can also masquerade as epileptic seizures. Structural heart issues such as cardiomyopathies, aortic stenosis, and other heart rhythm disorders, including tachyarrhythmias, atrioventricular conduction block, and inherited channelopathies, such as Brugada syndrome and catecholaminergic polymorphic ventricular tachycardia (CPVT), can cause syncopal episodes [12]. Thus, thorough cardiac examination including a 12-lead ECG is essential in assessing patients with syncope or seizure-like episodes.

In this case, there are some atypical features that are unusual for LQTS, including mild left ventricular dysfunction and T wave inversion [1]. Given these atypical features, a CMR study has been requested to exclude subtle structural heart disease, in particular arrhythmogenic cardiomyopathy.

## Conclusions

Thorough cardiac evaluation, including a detailed family history and 12-lead ECG, is essential for distinguishing between neurological and cardiac causes of syncope or convulsive movements. This case highlights the necessity for clinicians to maintain a high index of suspicion for cardiac disorders in patients with unexplained neurological symptoms, ultimately ensuring timely and effective care for those at risk of life-threatening arrhythmias. Twelve-lead ECGs should be done in all patients presenting with syncopal episodes. Increased awareness and education about LQTS and other cardiac causes of syncope among healthcare providers can facilitate better recognition and management of these potentially fatal conditions.
